# Analysis of whole-blood antioxidant capacity after chronic and localized irradiation using the i-STrap method

**DOI:** 10.1093/jrr/rrab099

**Published:** 2021-10-27

**Authors:** Lue Sun, Yohei Inaba, Yu Sogo, Kumi Morikawa, Naoki Kunugita, Koichi Chida, Takashi Moritake

**Keywords:** antioxidant, chronic irradiation, partial body irradiation, radiation disaster, emergency medicine

## Abstract

Ionizing radiation exposure affects the redox state *in vivo*. Recently, whole-blood antioxidant capacity (WBAC) has been reported to decrease in a dose-dependent manner after acute total body irradiation (TBI). However, changes in WBAC after localized and chronic irradiations have not been reported. This study analyzed changes to WBAC in mice after either localized irradiation (irradiation of the left hind leg only) or chronic TBI using the i-STrap method. Leg-localized irradiation exerted limited effects on WBAC, while WBAC decreased in a dose rate-dependent manner after TBI. Further, the WBAC reached the minimum value in a shorter period at a smaller dose rate. Our results suggest that changes in WBAC do not directly reflect absorbed dose, but may reflect radiation-induced biological damage at the systemic level. This study will contribute to the understanding of radiation-induced injuries and diseases, and will facilitate the establishment of biomarkers for radiation exposure.

## INTRODUCTION

In large-scale radiation accidents and/or disasters, biodosimetry offers a useful tool for estimating exposure doses in victims. Several methods of biodosimetry have been reported and used in radiation accidents, including chromosomal aberration [1] and DNA damage analysis [2].

Ionizing radiation exposure is known to induce oxidative stress in humans and animals, and Navarro *et al.* showed that the ratio of reduced/oxidized glutathione in blood was decreased by radiotherapy in cancer patients [3]. Further, radiation-induced lung and skin injuries are mediated by the induction of oxidative stress [4, 5]. In an analysis of whole-blood antioxidant capacity (WBAC) after acute total body irradiation (TBI) using the i-STrap method, Sun *et al.* revealed WBAC decreased in a dose-dependent manner [6, 7]. They suggested that WBAC may offer a potent biomarker for estimating exposure doses during nuclear and/or radiation accidents [6]. Not only acute total body exposure, but also localized and/or chronic exposure occurs in radiation accidents. However, previous reports have not investigated changes in WBAC caused after localized and chronic exposures.

The present study analyzed changes to WBAC in mice after either localized irradiation (irradiation of the left hind leg only) or chronic TBI. We found that localized irradiation ≥20 Gy decreased WBAC, but this may have been due to incomplete shielding of other body parts. Further, we found that WBAC decreased after TBI in a dose rate-dependent manner. Interestingly, the WBAC reached the minimum value in a shorter period at a smaller dose rate. Our results indicated that decreases in WBAC after radiation exposure were affected by irradiated area and dose rate.

## MATERIALS AND METHODS

### Mice and blood sampling

Six-week-old male C57BL/6 J mice were obtained from Japan SLC (Shizuoka, Japan). Both diet (MF diet; Oriental Yeast Co., Tokyo, Japan) and drinking water were sterilized by autoclaving and supplied ad libitum. Mice received at least 7 days of acclimation before receiving irradiation. Whole blood (100 μL) was collected from mice by puncture of the submandibular vein with a 0.5-mm Goldenrod Animal Lancet (MEDIpoint, New York, USA). The collection tubes were treated with heparin (FUJIFILM Wako Pure Chemical Corporation, Osaka, Japan). To avoid the risk of change in antioxidant capacity caused by repeated blood sampling, appropriate interval for recovery of mice was set for the blood collections in this study [8].

### Localized X-irradiation

**Fig. 1:**
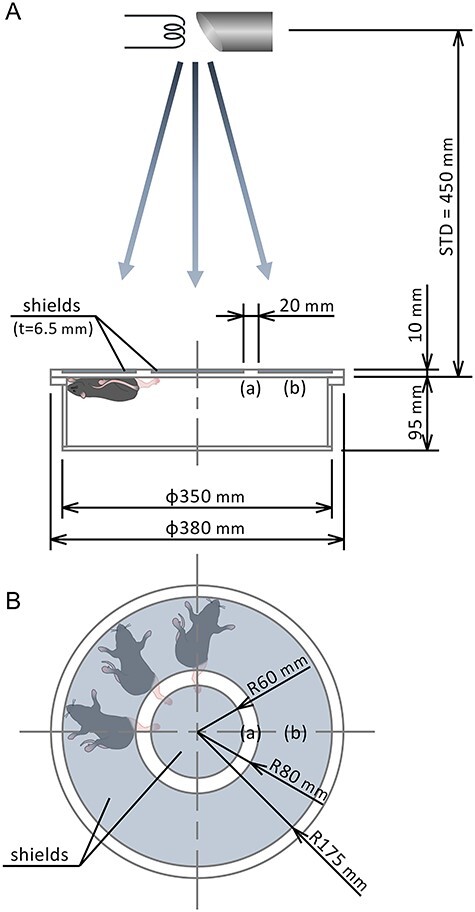
The custom-made collimator for localized irradiation. (A) Side view and (B) top view. Position of the radio-photoluminescent glass dosimeters for dosimetry: (a) non-shielded area; and (b) shielded area. Source-target distance (STD) is 450 mm. The shield is a stack of 3-mm brass, 3-mm Al, and 0.5-mm Pb.

Mice left hind leg underwent depilation using hair clipper and localized radiation using an X-ray generator (150 kVp; 20 mA; filter: 0.2 mm Cu and 0.5 mm Al; MBR-1520R-3; Hitachi Power Solutions, Ibaraki, Japan) with a custom-made collimator to expose only the left hind leg (Fig. 1). Mice were anesthetized during radiation exposure using three types of mixed anesthetic agents (0.75 mg/kg of medetomidine [Nippon Zenyaku Kogyo Co., Fukushima, Japan], 4.0 mg/kg of midazolam [Maruishi Pharmaceutical. Co., Osaka, Japan], and 5.0 mg/kg of butorphanol [Meiji Seika Pharma Co., Tokyo, Japan]). In addition, 0.75 mg/kg of atipamezole (Nippon Zenyaku Kogyo Co.) was used to reverse the effects of medetomidine after radiation exposure.

### Total body γ-irradiation

Acute (0.65 Gy/min = 936 Gy/day) or chronic (0.3 or 1 Gy/day) TBI was performed using ^137^Cs γ-rays. Total dose was 3 Gy for all dose rate groups. Mice did not receive anesthesia and were provided with ad libitum access to diet and water during exposure.

### Dosimetry

Absorbed dose was measured using a radio-photoluminescent glass dosimeter (GD-352 M; Chiyoda Technol Co., Tokyo, Japan). The shielding efficiency of the collimator was calculated from the ratio of dose rate in the shielding area to that in the non-shielding area (Fig. 1).

### Measurement of mouse skin reactions

Acute skin reaction after leg-localized irradiation was scored every 2–7 days for 80 days using an arbitrary scale (Table 1) [9].

**Table 1:** Score table for mouse skin reactions

Score	Observation
0	No abnormality
0.5	50/50 doubtful if there is any difference from normal
0.75	Slight but definite abnormality
1	Definite abnormality with reddening
1.25	Severe reddening and/or white scales and/or puffiness
1.5	Moist breakdown in one very small area with scaly or crusty appearance
1.75	Moist desquamation in small areas (more definite than 1.5)
2	Breakdown of large area, possibly moist in places
2.5	Breakdown of large areas of skin with definite moist exudate
3	Breakdown of most skin with moist exudate
3.5	Complete moist breakdown of limb—often stuck to body

Modified from the report by Iwakawa *et al.* [9]

### Measurement of WBAC (i-STrap method)

WBAC was measured using i-STrap (Dojindo/Dojin Glocal, Kumamoto, Japan), according to the protocol provided by the manufacturer [7]. Briefly, 100 μL of whole blood, 100 μL of saline, 10 mM of 2-diphenylphosphinoyl-2-methyl-3,4-dihydro-2H-pyrrole N-oxide (DPhPMPO), and 10 mM of tert-butyl hydroperoxide (tBuOOH) were mixed and incubated at room temperature for 30 min. In this step, tBuOOH reacted with hemoglobin in blood to produce organic radicals (tert-butyl, tert-butyloxyl, and tert-butylperoxyl radicals) through the Fenton reaction. These radicals competitively reacted with antioxidants in blood or DPhPMPO. After the incubation, DPhPMPO was extracted using 1 mL of chloroform/methanol (2:1) solution (FUJIFILM Wako) and measured by X-band ESR spectroscopy (JES-TE200; JEOL, Tokyo, Japan). The signal of DPhPMPO spin adduct intensity was corrected by marker manganese oxide intensity. When the blood contained smaller amounts of antioxidants (low blood antioxidant capacity), more radicals were trapped by DPhPMPO, and a higher ESR signal intensity was measured.

### Statistical analysis

The number of mice in each group is shown in Supplementary Table 1. Mean and standard deviation (SD) were calculated for each data point and normalized by the data of control group (corresponding 0-Gy group at each time point). Two-way analysis of variance (ANOVA) and the post hoc Dunnett’s test were used to determine the significance of differences from the control group. Values of *P* < 0.05 were considered significant.

### Ethical considerations

All animal experiments were performed in accordance with the Animal Care Guidelines of the University of Occupational and Environmental Health, Japan (UOEH.J.) and the National Institute of Advanced Industrial Science and Technology (AIST). All animal husbandry procedures and experiments were approved by the Animal Experiment Committee of UOEH.J. (permit number: AE15–009) and AIST (permit number: 2019–0349).

## RESULTS

### Shielding efficiency in the custom-made collimator

In the custom-made collimator, the dose rate in the non-shielded area (left hind leg of the target mouse) was 0.82 Gy/min, while that in the shielded area (mouse body) was 0.029 Gy/min. The shielding efficiency was thus 96.5%.

### Changes in WBAC after localized irradiation to the leg

**Fig. 2:**
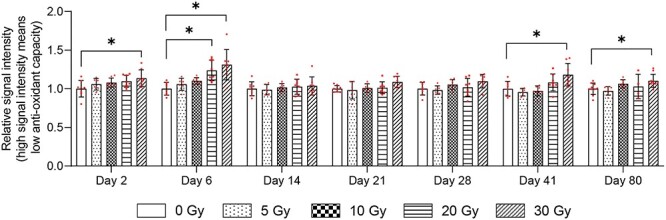
Changes in WBAC after localized irradiation to the left hind leg. WBAC was measured using i-STrap. Antioxidant capacity correlated inversely with signal intensity. Data were normalized by the data of control group (corresponding to the 0-Gy group at each time point). Bars indicate means, error bars indicate SDs, and red dots indicate individual data points. ^*^P < 0.05, ^**^P < 0.01, ^***^P < 0.001, ^****^P < 0.0001, two-way ANOVA and the post hoc Dunnett’s test were used to analyze significant differences from the control group.

**Fig. 3:**
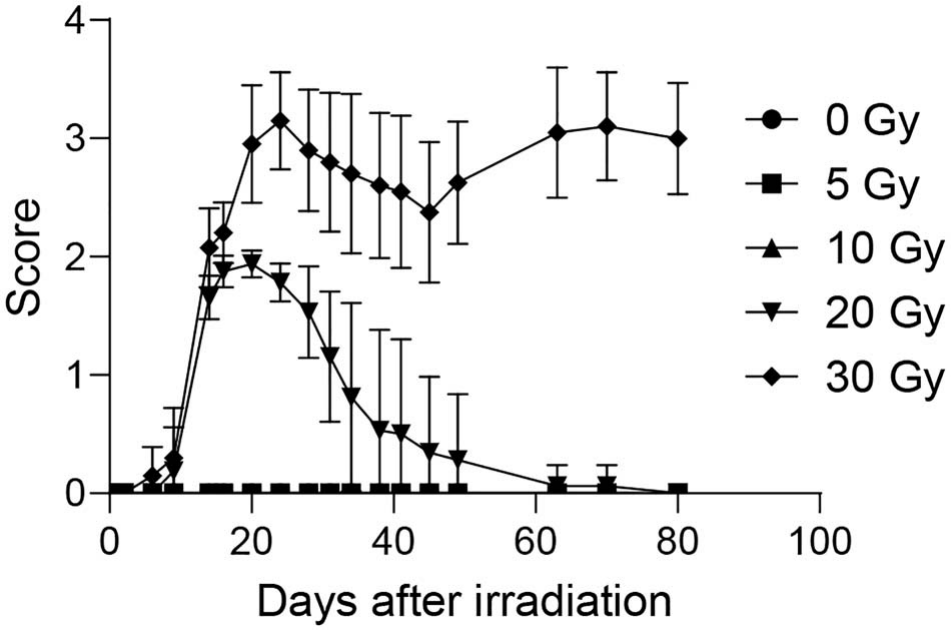
Changes in mouse leg skin reaction after localized X-irradiation. Symbols indicate means and error bars indicate SDs.

Neither 5- nor 10-Gy irradiation groups showed any changes in WBAC after irradiation (Fig. 2). The 20-Gy irradiation group showed a significant decrease in WBAC on Day 6 (Fig. 2). The 30-Gy irradiation group showed significantly decreased WBAC on Days 2, 6, 41 and 80 (Fig. 2). We found that ≥20-Gy irradiation induced skin injuries; by Day 80 after irradiation, injuries in the 20-Gy irradiation group had resolved, but those in the 30-Gy irradiation group had not (Fig. 3).

### Changes in WBAC after chronic TBI

**Fig. 4:**
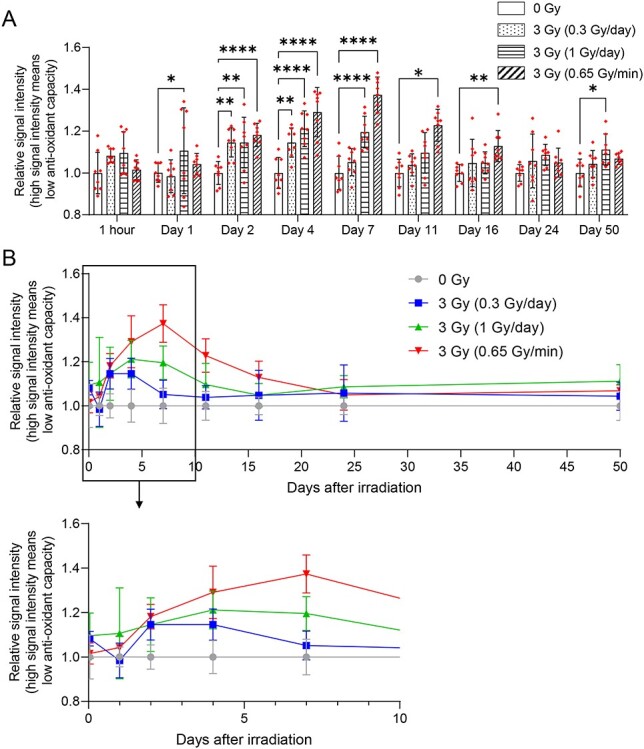
Changes in WBAC after acute or chronic TBI. Data are shown by (A) a bar graph for comparing normalized antioxidant capacities among all the irradiation groups at each time point and (B) a line graph for exhibition of time course change in normalized antioxidant capacity for each irradiation group. WBAC was measured using i-STrap. Antioxidant capacity correlated inversely with signal intensity. Data were normalized by the data of control group (corresponding to the 0-Gy group at each time point). Bars indicate means, error bars indicate SDs, and red dots indicate individual data points in the bar graph (A). ^*^P < 0.05, ^**^P < 0.01, ^***^P < 0.001, ^****^P < 0.0001, two-way ANOVA and the post hoc Dunnett’s test were used to analyze significant differences from the control group.

A schema of the experimental schedule is shown in Supplementary Fig. 1, and the results are shown in Fig. 4. We found that 3-Gy acute irradiation (0.65 Gy/min) significantly decreased WBAC from 2 to 16 days after irradiation and the lowest level was observed on Day 7. These results were consistent with a previous report from Sun *et al.* [6]. Irradiation at 1 Gy/day for 3 days (total dose, 3 Gy) resulted in significantly decreased WBAC on Days 1–7 after irradiation, with the peak decrease around Day 4–7. Irradiation at 0.3 Gy/day for 10 days (total dose, 3 Gy) resulted in significantly decreased WBAC, with the lowest level observed on Days 2–4 after irradiation. Furthermore, the level of decrease in WBAC seemed dependent on dose rate.

## DISCUSSION

Radiation exposure-associated changes in blood oxidative stress have been well-investigated. However, plasma or serum samples have been analyzed most often, probably due to the simplicity of collecting and examining these samples [10, 11]. Meanwhile, blood cells (particularly red blood cells) have active metabolism supporting homeostasis and function. The resulting metabolites may reflect health status or environmental stressors differently than metabolites from plasma [12]. Sun *et al.* reported that WBAC offers a promising biomarker of radiation exposure [6]. Mechanism remains unclear, but WBAC may involve changes in metabolites in red blood cells [6, 13].

Medical radiation exposures (e.g. radiotherapy or interventional radiology) and radiation accidents sometimes induce skin injuries [14–16]. Radiation-induced skin injuries have been well-investigated in mouse experiments. Iwakawa *et al.* showed that <30-Gy irradiation caused radiation-induced skin injuries to a limited extent [9]. The present study showed that skin injuries caused by 20 Gy and 30 Gy of irradiation were reversible and irreversible, respectively, using the same mouse strain and scores applied in the study by Iwakawa *et al.* (Fig. 3). However, radiation sources differed markedly, with Iwakawa *et al.* using ^137^Cs γ-rays while we used 150-kVp X-rays. Since lower-energy photon beams reportedly show higher linear energy transfer and cell killing ability [17], the difference in radiation sources may have contributed to the differences in results between the previous investigation [9] and the present study.

This study showed that 20 and 30 Gy of localized irradiation decreased WBAC (Fig. 2). However, the shielding efficiency was 96.5% in our experiments, so the mice body still received 0.7 or 1.05 Gy in the 20- and 30-Gy localized irradiation groups, respectively. A previous report showed that 0.5 Gy of TBI could decrease WBAC [6]. We thus considered that localized irradiation to the leg resulted in limited changes to WBAC, suggesting that WBAC may reflect radiation-induced damage at the systemic level rather than that localized to the leg. Further study should be performed to clarify whether localized irradiation to other parts of the body (e.g. head, chest, or abdomen) can induce a decrease in WBAC. Such research would help reveal the mechanisms underlying decreases to WBAC after radiation exposure.

We found that WBAC decreased after TBI in a dose rate-dependent manner (Fig. 4). The dose rate effect of radiation has been well-investigated. For example, Mitchel *et al.* showed that mice irradiated with a high dose rate (0.5 Gy/min) showed a shorter life span than those irradiated with a low dose rate (0.5 mGy/min) at the same total dose [18]. Amundson *et al.* also showed that acute irradiation induced much more cell apoptosis than chronic irradiation [19]. Our results were consistent with these results, suggesting that changes in WBAC did not directly reflect the absorbed dose, but may instead reflect radiation-induced systemic biological damage. Interestingly, the WBAC reached the minimum value within a shorter period at a lower dose rate (Fig. 4B). Changes in cell cycle distribution and DNA damage repair response reportedly occur during exposure [20, 21], suggesting that the process of WBAC decrease and recovery might occur during chronic exposure. Further studies are needed to reveal whether WBAC levels change during irradiation and whether WBAC changes in a total dose-dependent manner. Such research would help clarify WBAC responses to chronic irradiation.

Our results raise additional questions. We used young (~7 week old) male mice for analysis, but whether age, sex, or other lifestyle factors (diet, smoking, etc.) affect WBAC levels remains unclear. These data are important for determining the utility of WBAC as a biodosimeter for radiation exposure. Further studies should be investigated these points not only in mice, but also in human samples.

## CONCLUSION

The present study investigated WBAC changes after localized acute irradiation and chronic TBI. Unexpectedly, our results suggested that localized leg irradiation is unlikely to alter WBAC. We found that TBI decreased WBAC in a dose rate-dependent manner, suggesting that changes in WBAC may be associated with radiation-induced systemic biological damage. Further investigations are needed to elucidate the precise mechanisms underlying changes in WBAC after irradiation, and to determine whether WBAC would offer a useful biomarker of radiation exposure in humans and be causally related to radiation-induced injuries and diseases.

## Supplementary Material

Supplementary_fig_1_rrab099

Supplementary_table_1_rrab099
