# Supplementary figures and images for: Analysis of whole-blood antioxidant capacity after chronic and localized irradiation using the i-STrap method
Source: J Radiat Res. 2021 Oct 27;63(1):30–5. doi: 10.1093/jrr/rrab099 (PMC8776686; doi:10.1093/jrr/rrab099)

Supplementary fig. 1

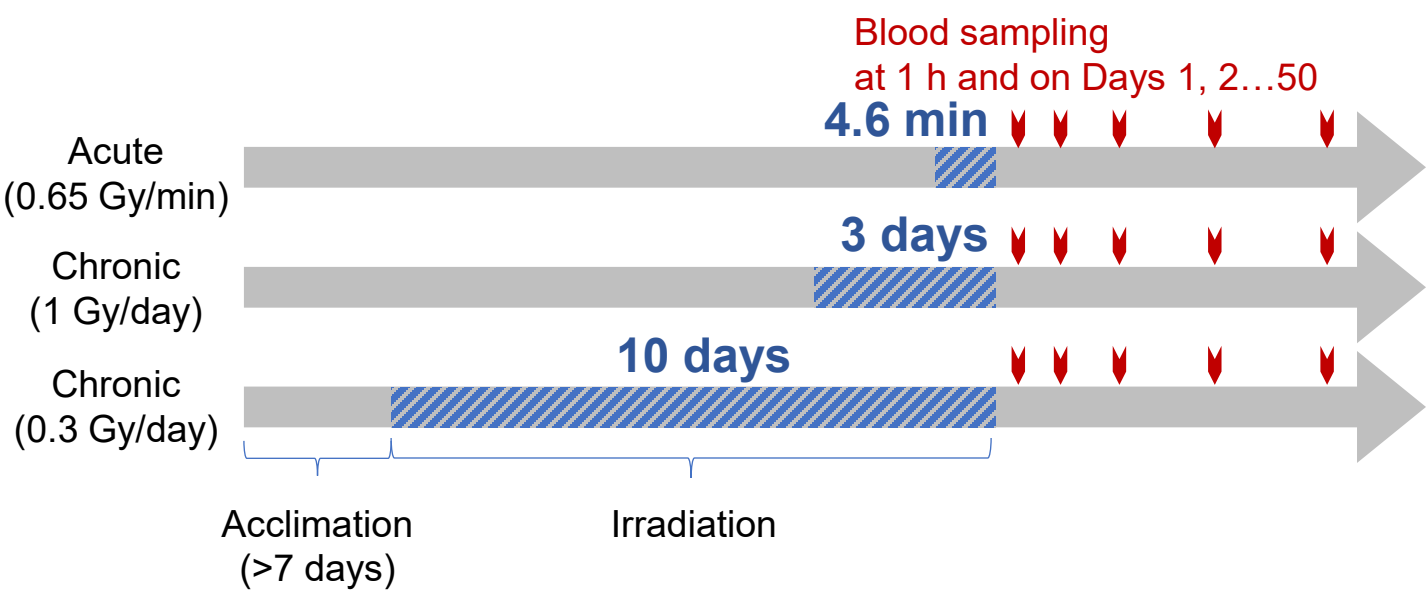

Supplement: Supplementary_fig_1_rrab099 [file supplementary_fig_1_rrab099.pdf]
